# A critical synthesis of literature on the promoting action on research implementation in health services (PARIHS) framework

**DOI:** 10.1186/1748-5908-5-82

**Published:** 2010-10-25

**Authors:** Christian D Helfrich, Laura J Damschroder, Hildi J Hagedorn, Ginger S Daggett, Anju Sahay, Mona Ritchie, Teresa Damush, Marylou Guihan, Philip M Ullrich, Cheryl B Stetler

**Affiliations:** 1 Northwest HSR&D Center of Excellence, VA Puget Sound Healthcare System, Seattle, Washington, USA; 2 Department of Health Services, University of Washington School of Public Health, Seattle, Washington, USA; 3 HSR&D Center for Clinical Management Research and Diabetes QUERI, VA Ann Arbor Healthcare System, Ann Arbor, Michigan, USA; 4 VA Substance Use Disorders Quality Enhancement Research Initiative, Minneapolis VA Medical Center, Minneapolis, Minnesota, USA; 5 Department of Psychiatry, School of Medicine, University of Minnesota, Minneapolis, Minnesota, USA; 6 VA Stroke QUERI, HSR&D Center of Excellence, Richard L. Roudebush VA Medical Center, Indianapolis, Indiana, USA; 7 Chronic Heart Failure QUERI Center, VA Palo Alto Health Care System, Palo Alto, California, USA; 8 Mental Health Quality Enhancement Research Initiative, Central Arkansas Veterans Healthcare System, North Little Rock, Arkansas, USA; 9 Indiana University Center for Aging Research, Regenstrief Inc., Indianapolis, Indiana, USA; 10 Spinal Cord Injury QUERI Research Coordinating Center, Center for Management of Complex Chronic Care (CMC3), Edward Hines, Jr. VA Hospital, Hines, Illinois, USA; 11 Spinal Cord Injury QUERI, VA Puget Sound Health Care System, Seattle, Washington, USA; 12 Independent Consultant, Amherst, Massachusetts, USA; 13 Health Services Department, Boston University School of Public Health, Boston, Massachusetts, USA

## Abstract

**Background:**

The Promoting Action on Research Implementation in Health Services framework, or PARIHS, is a conceptual framework that posits key, interacting elements that influence successful implementation of evidence-based practices. It has been widely cited and used as the basis for empirical work; however, there has not yet been a literature review to examine how the framework has been used in implementation projects and research. The purpose of the present article was to critically review and synthesize the literature on PARIHS to understand how it has been used and operationalized, and to highlight its strengths and limitations.

**Methods:**

We conducted a qualitative, critical synthesis of peer-reviewed PARIHS literature published through March 2009. We synthesized findings through a three-step process using semi-structured data abstraction tools and group consensus.

**Results:**

Twenty-four articles met our inclusion criteria: six core concept articles from original PARIHS authors, and eighteen empirical articles ranging from case reports to quantitative studies. Empirical articles generally used PARIHS as an organizing framework for analyses. No studies used PARIHS prospectively to design implementation strategies, and there was generally a lack of detail about how variables were measured or mapped, or how conclusions were derived. Several studies used findings to comment on the framework in ways that could help refine or validate it. The primary issue identified with the framework was a need for greater conceptual clarity regarding the definition of sub-elements and the nature of dynamic relationships. Strengths identified included its flexibility, intuitive appeal, explicit acknowledgement of the outcome of 'successful implementation,' and a more expansive view of what can and should constitute 'evidence.'

**Conclusions:**

While we found studies reporting empirical support for PARIHS, the single greatest need for this and other implementation models is rigorous, prospective use of the framework to guide implementation projects. There is also need to better explain derived findings and how interventions or measures are mapped to specific PARIHS elements; greater conceptual discrimination among sub-elements may be necessary first. In general, it may be time for the implementation science community to develop consensus guidelines for reporting the use and usefulness of theoretical frameworks within implementation studies.

## Background

Only a small proportion of research findings are widely translated into clinical settings [1], often due to barriers in the local setting [2]. The Promoting Action on Research Implementation in Health Services framework, or PARIHS, is a conceptual framework that posits key, interacting elements that influence successful implementation of evidence-based practices (EBPs) [3-7]. Implementation researchers have widely cited PARIHS or used it as the basis for empirical work [8-11]. This body of research has occurred against the backdrop of broad calls to incorporate theoretical frameworks in quality improvement implementation activities and research [12-14].

It has been over a decade since Kitson and colleagues first described the PARIHS framework, and while several papers have been published that update and propose refinements [4-7,14,15], there has not yet been a literature review to examine how the framework has been used in implementation projects and research. Our interest in PARIHS grew out of its use by numerous researchers involved in the Veterans Health Administration (VA) Quality Enhancement Research Initiative and their expressed need for guidance in how to use it in implementation projects. The purpose of the present article is to critically review and synthesize the conceptual and empirical literatures on PARIHS to: understand how PARIHS has been used; understand how its elements and sub-elements have been operationalized; and highlight strengths and limitations of PARIHS relative to use of the framework to guide an implementation study. We close with a set of recommendations to increase the value of the PARIHS framework for guiding implementation activities and research.

### PARIHS framework

PARIHS outlines the determinants of successful implementation of evidence into practice. It was initially published in 1998 as an unnamed framework inductively developed based on the experience of the authors with practice improvement and guideline implementation efforts [3]. They presented three case examples to illustrate its usefulness with accompanying descriptive analyses. Subsequently, two concept analyses were published exploring the maturity, meaning, and characteristics of facilitation [4] and context [5] as they relate to implementation. These concept analyses were based on non-systematic reviews of the literature. The original authors published a refined version of the framework in 2002 based on theoretical insights from these concept analyses [15]. This article contained the first published use of the PARIHS label. A conceptual exploration of evidence was published in 2004, which rounded out the PARIHS team's review of their framework's three core elements [6]. Kitson and colleagues published a further clarification of PARIHS in 2008. This latest paper proposed that PARIHS is best used in a two-step process: as a framework to diagnose and guide preliminary assessment of evidence and context, and to guide development, selection, and assessment of facilitation strategies based on the existing evidence base and local context [7].

**Figure 1:**
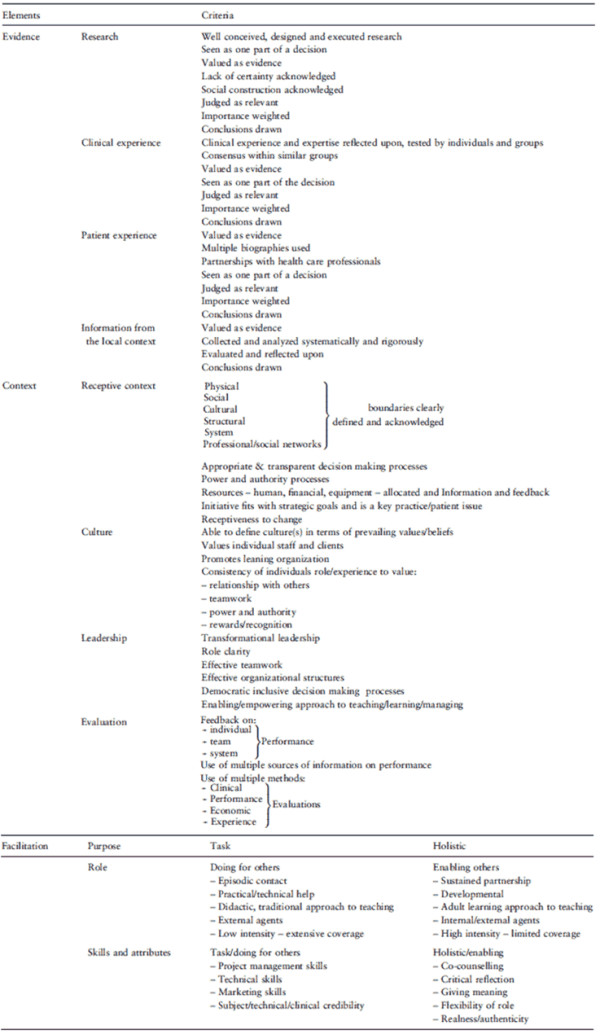
**Key elements for implementing evidence into practice, from Rycroft-Malone *et al. ***[29].

The framework comprises three, interacting core elements: evidence (E) - 'codified and non-codified sources of knowledge' [7] as perceived by multiple stakeholders; context (C) - the quality of the environment or setting in which the research is implemented; and facilitation (F) - a 'technique by which one person makes things easier for others,' achieved through 'support to help people change their attitudes, habits, skills, ways of thinking, and working' [3]. The core assertion is that successful implementation is a function of E, C, and F and their interrelationships. The status of each of these elements can be assessed for whether it will have a weak ('low' rating) or strong ('high' rating) effect on implementation (Figure 1).

In the PARIHS framework, evidence consists of four sub-elements, corresponding to four main sources of evidence: research evidence from studies and clinical practice guidelines including, but not limited to, formal experiments; clinical experience or related professional knowledge; patient preferences and experiences; and locally derived information or data, such as project evaluations or quality improvement initiatives [6,7]. A fundamental premise of PARIHS is that while research evidence is often treated as the most heavily weighted source, all four sources have meaning and constitute evidence from the perspective of end users.

Context comprises four sub-elements: receptive context, organizational culture, leadership, and evaluation [5,7]. All four of these sub-elements are defined in PARIHS core papers [5,7], and, for culture, leadership. and evaluation, definitions from the broader literature are cited in a related concept analysis [5]. For example, culture is alternatively described as a 'paradigm,' as '`the way things are done around here' and as a metaphor for the organization-something the organization is rather than something it possesses; leadership is described as an indicator or reflection of the 'nature of human relationships' in the organization, pertaining to the types of leadership roles enacted and who enacts them [3,5]; and evaluation is described largely in terms of feedback [5] and how performance data are collected and reported [7]. Descriptions of the sub-elements for each are provided in earlier papers that reflect 'high' and 'low' ratings that indicate a more or less favorable context for successful implementation, respectively. Indications for high ratings of context include, for example: clearly defined and acknowledged physical, social, cultural, structural and/or system boundaries; valuing individual staff and clients; promoting organizational learning; existence of transformational leadership as well as democratic or inclusive decision making; and existence of feedback on individual, team, and/or system performance [15,16].

Facilitation includes three sub-elements and an array of mechanisms to influence implementation of evidence into clinical practice. The first sub-element of facilitation focuses on its purpose; *e.g.*, whether facilitation is to support attainment of a specific goal (task-oriented) or enable individuals or teams to reflect on and change their attitudes and ways of working (holistic-oriented) [15]. In the PARIHS framework, these two purposes are arrayed as endpoints on a continuum. The second and third sub-elements of facilitation are the role of the facilitator(s) and their associated skills and attributes, which are described for each of the two purposes. On the task-oriented end of the continuum, the facilitator might engage in episodic contacts and provide practical focused help, which requires strong project management/technical skills but a relatively low level of intensity. On the holistic-oriented end of facilitation, the facilitator might focus on building sustained partnerships with teams to assist them in developing their own practice change skills. This requires a relatively high level of intensity.

## Methods

We used qualitative, critical synthesis methods for this review because our objectives were descriptive (*e.g.*, describing how PARIHS has been used) and critical (*e.g.*, appraising relative strengths and weaknesses of the framework), rather than meta-analytic (*e.g.*, calculating an average effect size) [17]. We describe our review process below.

### Search strategy and selection of publications

Our literature search included three sources. First, we conducted key word searches of the PubMed and CINAHL databases using the terms 'PARIHS' and 'promoting action on research implementation in health services.' We selected PubMed because it represents the preeminent database of peer-reviewed literature in the health fields, and CINAHL because it focuses specifically on nursing literature, where some of the original PARIHS concept papers were published. We used limited key words because this review was focused on the PARIHS model, rather than implementation models generally. Second, we reviewed the reference lists of included articles. Third, we solicited citations from a PARIHS author and other colleagues familiar with this body of research.

We selected articles based on four *a priori *criteria: published peer-reviewed literature, English language, published prior to March 2009, and explicit reference to the PARIHS framework either by name or citation of core conceptual articles. We did not specify *a priori *exclusion criteria.

### Appraisal and abstraction of articles

We appraised and abstracted include articles in a three-step process. First, each article was read by a primary reviewer who wrote a narrative synopsis using a template (see Additional File 1, Synopsis template). The purpose of the initial synopsis was to provide an overall summary and critique of the article. Second, the completed synopsis was distributed and reviewed by all co-authors, and discussed and refined on a conference call. Third, one of the co-authors condensed each synopsis using a structured summary table, with a separate table for each article. The purpose of the summary tables was to create a concise, structured appraisal and critique for each article. Some papers were empirical and others were conceptual. Summary tables for empirical articles included the overall method/design, an appraisal of study quality, study outcomes, how PARIHS was proposed to be used and actually used, and assessment of congruency between PARIHS and study methods (see Additional File 2, Empirical article summary table). These tables also listed how PARIHS elements and sub-elements were defined and measured or operationalized in the study, along with findings, barriers, and enablers to implementation. The summary tables for core concept articles focused on the framework's elements, sub-elements, limitations, recommendations, and other observations (Additional File 3, Core-concept article summary table). These summary tables were reviewed by the primary reviewer for that paper and again by all co-authors, discussed as a group, and affirmed or revised as needed. This collection of empirical and core summary tables constituted the analytic foundation for our meta-summary and synthesis.

### Meta-summary and synthesis

Four co-authors reviewed the final set of summary tables and independently highlighted key points per article to create a meta-summary. Key points represented concepts, specific findings related to PARIHS generally and/or to specific elements or sub-elements, observations about the use of the framework, and conclusions. Information highlighted as a key point by at least three of the four co-authors was discussed further at a two-day, in-person working conference. The purpose of the discussion of key points was to explore and summarize similarities and differences across the papers (both empirical and core conceptual) and to develop qualitative themes. Some of the themes were descriptive, *e.g.*, regarding the actual versus articulated use of PARIHS. Other themes were interpretive, *e.g.*, our consensus judgments regarding overall limitations, related issues, and strengths of the framework relative to the ability of researchers to effectively use it to guide an implementation study. We developed implications for using the framework as well as related recommendations based on these synthesized findings. As with the article appraisal, the synthesis and recommendations were discussed with all co-authors and refined until consensus was reached.

## Results

### Search results

**Figure 2:**
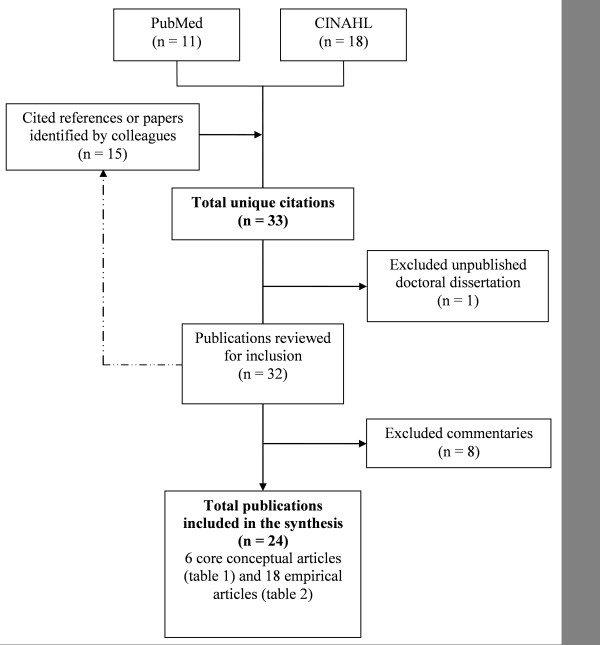
**Flow diagram of literature review **.

We initially identified 33 unique articles (Figure 2). We excluded an unpublished doctoral dissertation [18], and eight commentaries [19-26]. Commentaries did not reflect planned or actual application or refinement of PARIHS (See Additional File 4, Table of commentaries excluded from the synthesis). We included the remaining 24 articles in our review.

We characterized six articles as core concept articles (Table 1 Overview of core concept articles for the PARIHS framework). These were written by members of a PARIHS coordinating group (http://www.PARIHS.org/pages/contact_us.html) for the stated purpose of introducing [3] or elaborating on the framework, either as a whole [7,15], or on one of its three core elements [4-6]. The remaining 18 articles (Table 2 Overview of empirical articles included in the synthesis) were a mix of case reports and qualitative or mixed-methods studies [27-33], quantitative studies [9-11,34-36], literature reviews [37-39] and study protocols [40] or frameworks [41]. We refer to these collectively as empirical articles to distinguish them from the core concept articles.

**Table 1:** Overview of core concept articles for the PARIHS framework

Author	Year	Journal	Method	Sample	Focus of paper
Kitson	1998	Qual Health Care	Conceptual	Not applicable	Original paper proposing the framework (later named PARIHS) in which core elements are defined.

Harvey	2002	J Adv Nurs	Concept analysis	95 articles and books published 1985 - 1998, identified from Medline, Cinahl, Pyschlit or Sociofile.	Explore maturity of the concept of facilitation as part of on-going development/refinement of PARIHS.

McCormack	2002	J Adv Nurs	Concept analysis	Review of literature included 'seminal texts' and papers identified through Medline, Cinahl, Psychlit and Sociofile (search methods and details unclear).	Identify 'meaning, characteristics and consequences of practice contexts' as it relates to implementation. Part of on-going development/refinement of PARIHS.

Rycroft-Malone	2002	Qual Saf Health Care	Conceptual	Not applicable	Original authors present theoretical refinements to PARIHS framework, based on the concept analyses

Rycroft-Malone	2004	J Adv Nurs	Debate	Not applicable	'...aims to move on the debate...about the nature of evidence, describe the characteristics of evidence, and consider how different sources of evidence contribute to patient care.' Framed as a debate but part of on-going development/refinement of PARIHS.

Kitson	2008	Implement Sci	Conceptual	Not applicable	Provides a summary of the team's 'conceptual and theoretical thinking' and future directions for PARIHS, including items to operationalize PARIHS elements in the Appendix.

**Table 2:** Overview of empirical articles included in the synthesis

Author	Year	Journal	Method	Sample	Purpose of study/paper	Rationale for using PARIHS	How PARIHS was to be used/operationalized
Alkema	2006	Home Health Care Serv Q	Protocol	Not applicable.	Protocol for collecting qualitative data for translational study of medication management.	No explicit rationale.	Organizing framework for highlighting differences between efficacy studies and a planned translational study.

Bahtsevani	2008	J Eval Clin Pract	Quantitative survey development	2006 cross-sectional survey of 39 clinicians from 11 departments in academic hospital in Sweden.	Test-retest reliability of survey derived from PARIHS.	PARIHS implicitly presented as a validated explanatory framework.	As basis for a survey tool; items operationalized directly from Swedish translation of PARIHS sub-elements.

Brown	2005	Worldviews Evid Based Nurs	Lit review	Literature search was conducted using CINAHL and MEDLINE electronic databases reviewing studies from 1980 to 2004, yielding 90 papers. In addition, hand search yielded another 10 articles. 58 papers were chosen and read.	'Explore the factors that have a significant influence on getting evidence into practice ...and examine the relevance of these factors to postoperative pain practices' (p 131)	No explicit rationale but the authors state that PARIHS was used because translation is complex.	Organizing framework for assessing/analyzing studies that implemented pain management practices.

Conklin	2008	Can J Nurs Res	Mixed methods case study	Qualitative data from documentation and four telephone interviews, and survey completed by six Webcast participants from Canadian Seniors Health Research Transfer Network (SHRTN).	Evaluate performance of Ontario's Seniors Health Research Transfer Network for smoking cessation.	No explicit rationale.	Framework to evaluate a 'practical test' of the SHRTN network at three levels: Network-wide, Network component, and Implementation Site.

Cummings	2007	Nurs Res	Quantitative model	Cross-sectional survey of 6,526 nurses; 52.8% response rate, per secondary analysis of prior data (1998 Alberta Registered Nurse Study).	Develop and test theoretical model of organizational influences that predict RU by nurses and assess influence of context on RU.	PARIHS provides a framework to develop testable hypotheses about RU.	To map secondary data to components of context (culture, leadership, and evaluation) and facilitation.

Doran	2007	Worldviews Evid Based Nurs	Framework	Not applicable.	Create 'an outcomes-focused knowledge translation framework ... to guide the continuous improvement of patient care through the uptake of research evidence and feedback data about patient outcomes.'	No explicit rationale but said to be 'helpful in identifying the important elements within the practice setting that need to be in place in order to foster the uptake of evidence into practice'	As guide to develop their untested framework to enhance reflective professional practice generally; not applied to a specific implementation project.

Ellis	2005	Worldviews Evid Based Nurs	Case reports	Nurse managers (n = 16) from different locations in rural hospitals (n = 6) in Western Australia who participated in pre-workshop interviews; nurses who attended workshops and completed evaluation forms (n = 54); and nurses (n = 23) who participated in follow-up interviews.	Explore importance of context and facilitation in successful EBP implementation and foster EBP as a process.	PARIHS recognizes that implementing EBP relies on more than just the provision of best information.	As an organizing framework to code qualitative data and describe findings.

Estabrooks	2007	Nurs Res	Quantitative model	Cross-sectional survey of 4,421 nurses, nested within 195 specialty areas, nested within 78 acute care hospitals, per secondary analysis of prior data (1998 Alberta Registered Nurse Study).	To determine independent factors that predict research utilization among nurses, taking into account influences at individual nurse, specialty, and hospital levels.	PARIHS includes contextual factors.	To map secondary data to components of context (culture, leadership, and evaluation) and facilitation.

Meijers	2006	J Adv Nurs	Lit review	Articles from key word search of 5 databases (*e.g.*, CINAHL, Medline) through March 2005.	Systematic literature review exploring relationships between contextual factors and RU by nurses.	PARIHS includes contextual factors.	To map contextual factors from the literature.

Milner	2005	J Eval Clin Pract	Lit review	12 articles and 1 dissertation from 144 articles screened from search of major databases, *e.g., *CINAHL, Medline, PsycINFO (through Fall 2003), plus hand search of key journals.	Systematic literature review assessing factors affecting RU by 'clinical nurse educators.' Provide insight into usefulness of PARIHS 'as a conceptual framework to guide further study in the field.' p. 641.	PARIHS reflects the complexity of research implementation process, and specifically assesses facilitation as a distinct function.	As 'backdrop' to strengthen the analysis; to map findings.

Owen	2001	J Psychiatr Ment Health Nurs	Case report	Undisclosed number of sources of information, including staff from each service within a single specialist psychiatric service and female service users in the Rehabilitation and Community Care Service specialist services in United Kingdom.	Describe changes in specialist psychiatric services for women with serious, enduring mental problems.	No explicit rationale.	To 'plan, implement, monitor and evaluate the changes...' (p 226).

Rycroft-Malone	2004	J Clin Nurs	Qualitative	Focus groups (n = 2) to inform the development of an interview guide. Key informant interviews (n = 17) at two case study sites in United Kingdom.	Identify factors that practitioners deem most important to implementation and whether they match up with evidence, context and facilitation concepts.	PARIHS refinement by original authors.	To map identified factors.

Sharp	2004	Worldviews Evid Based Nurs	Qualitative	Clinical and non-clinical staff (n = 51) at United States Veterans Health Administration hospitals (n = 6) implementing changes in LDL-c (low-density lipoprotein cholesterol) screening and treatment. Interviews conducted between January and April 2001.	Identify barriers and facilitators to implementing strategies to improve measurement and management of LDL-c in coronary heart disease patients.	PARIHS includes contextual factors and facilitation in addition to evidence.	As an organizing framework for analysis of qualitative findings.

Stetler	2006	Implement Sci	Qualitative	United States Veterans Health Administration QUERI researchers (n = 7) from quality improvement/implementation projects (n = 6).	Exploration of facilitation in QUERI implementation projects.	Facilitation highlighted as 'theoretically-promising to the change agent role of QUERI' (p 2).	Used, as applicable, to help interpret identified thematic findings in this open-ended conceptual evaluation.

Wallin	2005	Int J Nurs Stud	Qualitative	Focus groups of intervention (n = 2) and control site (n = 2) teams from RCT at 4 county hospitals in central Sweden.	Explore perceptions and experiences of change teams and staff that had participated in an RCT regarding. Implementation of new neonatal guidelines.	PARIHS emphasizes interplay between evidence, context, and facilitation.	Used as an organizing framework to describe findings; also had used 'facilitation' and guidelines (evidence) as an intervention in the primary study.

Wallin	2006	Nurs Res	Quantitative model	Secondary analysis of two cross-sectional survey datasets (n = 504 and n = 5,946) (1996 & 1998 Alberta Registered Nurse Study).	Derive a measure of RU and validate the measure through 4 procedures.	PARIHS purported to be multi-dimensional, non-linear and includes variables other than individual characteristics and has been used in an increasing number of studies.	Responses to 3 items from the Alberta Registered Nurse survey that were deemed to best represent sub-elements of PARIHS context (culture, leadership, and evaluation) were used to group responses as having low, moderately low, moderately high, or high context to test whether RU is positively associated with context.

Wright McCormack*	2006 2007 2008	Nurs Older People Interna'l J Older People Nurs Unpublished Final Report	Quantitative case study & instrument development	Northern Ireland and Republic of Ireland. Multiple samples from multiple sites for case study and then tool development. *E.g.*, case study focus groups (n = 26 staff); and large sample validity study in Republic of Ireland location (n = 479) from 27 different sites.	Identify influence of contextual factors on evidence-based continence care in rehabilitation settings; and develop and conduct psychometric validation of a related Context Assessment Index (CAI) to enable practitioners in such settings to assess their context.	Not explicitly indicated but authors stated that the framework illustrates and makes sense of the complex factors involved in implementing evidence into practice.	To guide structure of study, based on constructs of culture, leadership and evaluation.

*We include a single entry for this project led by McCormack and McCarthy; this is the same project reported by Wright and colleagues in two articles: Wright, J. (2006). 'Developing a tool to assess person-centred continence care.' Nurs Older People**18**(6): 23-8; Wright, J., B. McCormack, et al. (2007). 'Evaluating the context within which continence care is provided in rehabilitation units for older people.' International Journal of Older People Nursing **2**(1): 9-19.

Two of the empirical articles reported on the same study in which the Context Assessment Instrument (CAI) was developed based on PARIHS [35,36]. We also obtained an unpublished final report for the project [42], which included all of the material in the two articles plus more methodological detail. We combined these sources into a single entry in Tables 3 and 4, yielding 17 study entries.

**Table 3:** Core concept articles

Author	Year	Journal	Strengths and issues re: PARIHS	Strengths and issues re: study/paper
Kitson	1998	Qual Health Care	Strengths: • PARIHS is described for the first time (but not yet named as such). It is an intuitively appealing framework that is succinct and yet allows for dynamic complexities of implementation. • Framework anticipates interrelationship among the three main elements. • PARIHS was an early well-articulated framework that went beyond focusing on evidence and acknowledged the non-linear nature of implementation. Issues: • Inconsistency in definitions and terms within the text of the article and terms presented in the table. • The defined continuums lacked consistency and valence (*e.g.*, 'low regard for individuals' on the 'low' end and 'patient centered' on the 'high' end) (p. 151). • Sources considered 'high' research evidence are limited. Culture seems to include everything and lacks clarity. Does not differentiate external facilitation versus internal facilitation (*e.g.*, through management or champions). Judges task-oriented facilitation as 'low' and 'holistic' facilitation as 'high.' Some concepts seem conflated (*e.g.*, receptive context includes 'inclusive decision-making processes' which seems equally related to sub-element of leadership). • Proposed as a diagnostic tool to help prepare the context and select the most appropriate intervention but supportive studies were limited and retrospective.	Strengths: • Theory paper that proposes PARIHS as an inductively developed framework to help understand complex implementations. Issues: • Rationale for mapping findings from sample studies into PARIHS elements is unclear and loose. For example, in one case, physicians rejected evidence-based guidelines and the authors attribute this to inadequate facilitation without clear rationale for the attribution.

Harvey	2002	J Adv Nurs	Strengths: • Authors suggested that there is some evidence that facilitators may help change clinical and organizational practice, although current data limited their ability to make conclusions. Issues: • Regardless of their suggested changes to the framework per the literature, the authors point out that further research is still needed on this inherent part of the framework, *i.e.*, regarding different models of facilitation. • Definitional clarity in related sub-elements remains an issue; and some promising, potential sub-elements identified in the paper did not make it into their suggested refinements.	Strengths: • Literature review included 'analysis of a broad range of health care literature.' (p. 579). Provided information on the level of maturity of the facilitation concept. • Provided information for model refinement. • Pointed out the need for more research on different models of facilitation; *e.g.*, the need to better differentiate external and/or internal facilitation. Issues: • Missing details about how the analysis was conducted, beyond authors' brief description of Morse (1995) and Morse et al.'s (1996) approach.

McCormack	2002	J Adv Nurs	Strengths: • Provided some substantiation of contextual elements; especially for holistic view of implementation. • Provided some conceptual backing. • Evolved a sub-element in context from measurement to evaluation. Issues: • Concept of context lacks clarity because of the many ways it is characterized; *e.g.*, 'what is clear from studies reviewed that have included a consideration of context is that there is inconsistency in the use of the term and that this has an impact on claims of its importance. Thus the implications of using context as a variable in research studies exploring research implementation are as yet largely unknown.' p. 101. • Muddles whether Context is an overarching element or a sub-element on equal footing with Culture, Leadership, Evaluation that needs to be subsumed under some other broader category: *i.e.*, 'the analysis of the characteristics and consequences of context suggests that other characteristics are equally important...and that these sub-elements need to be taken into account in any articulation of the concept of context.' p. 101.	Strengths: • Draws on broader literature addressing context. Issues: • Key details of methodology missing, including parameters such as years covered by search and numbers of articles reviewed and included. • Seemed to focus more on holistic organizational change versus task-oriented implementation.

Rycroft-Malone	2002	Qual Saf Health Care	Strengths: • Model now refined per concept analyses. • 'Its relative simplicity and intuitive appeal.' Issues: • Increased complexity of the framework; added sub-sub-elements; muddled the definitions in some cases, *e.g.*, with language such as social construction acknowledged vs. perhaps consensus determined (This may reflect cultural/language/philosophical differences) • Some clearly stated attributes of a facilitator were lost.	Strengths: • Responsive to their concept analysis work to further the theoretical development of the framework. • Recognizes that this is not a 'final' framework; noting that there will be continued evolution and 'it would be premature to suggest that this represents a final version' p. 178. Issues: • Did not delve into relationships among the core elements.

Rycroft-Malone	2004	J Adv Nurs	Strengths: • PARIHS' expansive acknowledgement of what can and should constitute 'evidence' in implementing EBPs Issues: • Sub-element definitions lack clarity • More understanding needed about how to integrate the multiple sources of evidence and how this melding can inform clinical decision-making	Strengths: • Tackling the issue of the nature of evidence versus traditional approaches. Issues: • Lack of clarity demonstrated when authors talk about testing their framework for 'patient-centered evidence-based care' (p. 87-8)

Kitson	2008	Implement Sci	Strengths: • Asserted that PARIHS can embrace multiple theories. • Further explored potential use of the model for a 'two-stage diagnostic and evaluative approach' focused on E and C whereby 'the intervention is shaped and moulded by the information gathered' in terms of the F element (p. 1-2). Issues (Appendix): • Lack of conceptual and definitional clarity of various items. Left the reader to figure out who the actor is; *e.g.*, 'the research evidence is of sufficiently high quality' begs the question, who is deciding and according to whose standards? (p. 1 of Additional File 1) • Phase 3's evolution lacked congruency with Phases 1 and 2, contributing to continued lack of consistency and definitional and conceptual clarity as one can't always see how a given phase builds to the next.	Strengths: • Appendix provided clearest guidance to date to define and operationalize sub-elements. Issues: • Not clear what main thesis or objective was; article appeared written with multiple objectives.

**Table 4:** Empirical articles

Author	Year	Journal	Strengths and issues re: PARIHS	Strengths and issues re: study/paper
Alkema	2006	Home Health Care Serv Q	Strengths: • As an organizing device for highlighting differences between intervention and implementation studies. Issues: • Variable interpretation of elements/sub-elements relative to the model, which implies its lack of definitional clarity and/or need for more direction in its application.	Strengths: • Novel in using the framework to highlight differences between original and translational trials. Issues: • Just a description of a protocol; no data.

Bahtsevani	2008	J Eval Clin Pract	Strengths: • Finds evidence of test-retest reliability for scale measuring PARIHS elements suggesting stability of constructs. Issues: • Item wording taken directly from Swedish translation of PARIHS, with some respondents confused about the meaning of related survey items. • Variable interpretation of PARIHS elements; *e.g.*, 'task-oriented' role was placed on the negative/low end of their rating scale.	Strengths: • One of only two articles included in the synthesis that attempts to develop an instrument based on PARIHS. Issues: • Only test-retest, and follow-up conducted after >4 weeks, too long for test-retest; categorical ratings were dichotomized to assess reliability with Kappa, instead of using a measure appropriate to categorical ratings.

Brown	2005	Worldviews Evid Based Nurs	Strengths: • Conclude that 3 PARIHS components apply very well to translation of pain management evidence into practice. Issues: • There appear to be 2 types of roles not differentiated in the model highlighted by this review: 1) Those in pre-existing roles, like clinical nurse specialists or nurse managers, which are a built-in facilitator as implementation/change may be an inherent part of what they do; 2) Someone on a project that is appointed to that interim role.	Strengths: • Systematic review. Issues: • Qualitative/observational review only, with no inclusion of interventional studies. • No data tables and lack of information re: methods for analysis and interpretation. • Focused on pain management literature which is very sparse.

Conklin	2008	Can J Nurs Res	Strengths: • Demonstrated flexible use of model whereby user chooses only those elements that applied to the target at hand, *i.e.*, levels of Networks. • Authors viewed findings as consistent with PARIHS, which emphasizes need for context-sensitive facilitation activities. • Results suggest that PARIHS has potential as a guide for evaluating other knowledge networks. Issues: • Highlighted the need to add focus on impacts or results to the framework. • Authors focused on understanding the knowledge exchange dimensions at the element level without exploring them at their sub-element level.	Strengths: • Explicitly defined outcomes as they relate to PARIHS. • The network level allowed context which can be seen as the resources or opportunities for effective communication and infrastructure opportunities like web cast. Issues: • Limited project with little data or clear logic for how results or conclusions were derived, and how the PARIHS elements were associated with the outcomes.

Cummings	2007	Nurs Res	Strengths: • Indirect support for facilitation being correlated with context. Higher RU and lower rate of adverse events associated with positive context (culture, leadership, evaluation). Issues: • 'Two unanticipated findings were that the concepts of innovation and facilitation had no significant influence on nurses' research utilization' (p S35).	Strengths: • One of only 2 studies that use quantitative models to test influence of specific context and facilitation measures on research utilization. Issues: • Variables loosely mapped to PARIHS along with other non-PARIHS variables. • Complex constructs measured using single-items that were selected post-hoc. RU (dependent variable) also calculated based, in part, on contextual variables (*e.g.*, autonomy, organizational slack). Authors note that perhaps facilitation was '... not operationalized ideally.'(p. S35)

Doran	2007	Worldviews Evid Based Nurs	Strengths: • 'The model is helpful in identifying the important elements within the practice setting that need to be in place in order to foster the uptake of evidence into practice. It shows that evaluation is an important component of the context for change and indicates that multiple methods and sources of feedback should be incorporated into an organization's evaluation framework.' (p. 4) • Authors operationalized all three main components of PARIHS - apparently choosing only sub-elements that seemed to apply to their objective. Issues: • '...previous descriptions of the model do not specifically address what indicators are appropriate for evaluating nursing systems and services or how to use performance measurement and feedback to design and evaluate practice change.'	Strengths: • Provides another example of the flexible and selective use of PARIHS and additional thoughts on the evaluation component. Issues: • Model yet to be applied/tested.

Ellis	2005	Worldviews Evid Based Nurs	Strengths: • Rationale for use based on: 'Embraced by academics, clinicians, and managers because it resonates with their own experience' (p. 85). • Supported PARIHS components; authors thought overall outcomes probably due to leadership, evidence, and facilitation and felt one of six hospitals did not implement due to 'clear' lack of leadership. Issues: • Noted by authors as not including underlying motivations (*e.g.*, relative advantage or dissatisfaction as tension for change) related to protocol/intervention. • Variable definitions of elements.	Strengths: • At least to some extent, assessed the nature of the framework and needs for refinement. Issues: • Low-level qualitative case study; some details of methods unclear (*e.g.*, what proportion of participating hospitals' nurses attended); convenience sample. • 'Many of workshop participants did not work in practice location...where the protocol was to be implemented... ' (p. 91).

Estabrooks	2007	Nurs Res	Strengths: • Facilitation, context (leadership, evaluation, and culture) were significant at the specialty level in addition to other contextual measures; *e.g.*, nurse-to-nurse collaboration (p. S7). Issues: • 'Variation in research utilization was explained mainly by differences in individual characteristics, with specialty- and organizational-level factors contributing relatively little by comparison...' (p. S7). • Results imply that PARIHS should be extended to include other contextual variables not explicitly included in the current version (*e.g.*, nurse-to-nurse collaboration).	Strengths: • One of only two studies that use quantitative models to test influence of specific context and facilitation measures on RU. • First demonstration of multi-level modeling approaches. Issues: • Variables loosely mapped to PARIHS along with other non-PARIHS variables. • Complex constructs were measured using single-items that were selected post-hoc. • RU (the dependent variable) is calculated based, in part, on contextual variables (*e.g.*, autonomy, organizational slack).

McCormack et al	2008	CAI Documents	Strengths: • Most comprehensive attempt to operationalize context CAI appeared to be successful for practitioners to generically reflect on their practice. • Provided useful information for potentially refining the framework in terms of enhancing the meaning of context. Issues: • Findings were said to suggest that some contextual characteristics are 'less theoretically robust than thought.' • Findings included 'factors' not consistent with the current structure of the four sub-elements under Context; variable placement of sub-sub-elements. • Tool seems to be especially useful for a holistic practice focus rather than for task-specific implementation.	Strengths: • Rigorous empirical development. Issues: • Need for further research regarding validity, reliability, and usability in other settings and with different clinical topics.

Meijers	2006	J Adv Nurs	Strengths: • In the literature, 'Six contextual factors were identified as having a statistically significant relationship with research utilization, namely the role of the nurse, multi-faceted access to resources, organizational climate, multifaceted support, time for research activities and provision of education' (p. 622). • 'The contextual factors could successfully be mapped to the dimensions of context in PARIHS (context, culture, leadership), with the exception of evaluation' (p. 622). • Authors 'believe that PARIHS is a fruitful starting point for better understanding of the impact of context on research utilization and more studies should explore this area of inquiry' (p. 632). Issues: • 'No single included study was assessed to be of high methodological quality' (p 626).	Strengths: • A comprehensive review of the literature. Issues: • The basis for mapping of contextual variables found in the literature onto the PARIHS framework was unclear.

Milner	2006	J Eval Clin Pract	Strengths: • Authors report general match of empirical findings to PARIHS. Issues: • Empirical findings didn't map to many sub-elements.	Strengths: • Systematic review with very thorough search strategy and clear inclusion/exclusion criteria. Issues: • Lack of clarity about how independent variables were measured (*i.e.*, how factors were to be mapped to PARIHS elements). • Focus seemed primarily on user's characteristics in general, not on role as an explicit facilitator, and not explicitly on successful implementation.

Owen	2001	J Psychiatr Ment Health Nurs	Strengths: • Used 1998 version of PARIHS but content highlighted in case study confirmed later PARIHS modifications: *i.e.*, use of evidence not just from RCTs (*e.g.*, from program eval), use of local data, and patient 'experiences.' • Brainstorming around E, C, and F seemed to illustrate dynamic interactions among these elements, as aspects of one were reflected in another. • Were able to use the framework to analyze their current situation. • Noted the importance of patient engagement. • Used along with other models of practice and evaluation. Issues: • Needs more emphasis in the model on 'motivating multi-disciplinary groups of staff to change and accept new ideas' (p 230). • Importance of patient engagement was highlighted but unclear if is part of both evidence and/or culture.	Strengths: • With open, albeit limited case study format, able to identify important 'additional' components beyond the cited 1998 model. Issues: • Lacks sufficient details about methods to evaluate changes, *e.g.*, re: services; source of recommendations; interviewees, data analysis or results.

Rycroft-Malone	2004	J Clin Nurs	Strengths: • They added 'fit' under context; *i.e.*, 'Initiative fits with strategic goals and is a key practice/patient issue' (p. 922). • Added 'Receptive' to sub-element of context; within that sub-element, added 'Resources - human, financial, equipment - allocated' as well as - 'Professional/social networks '(p. 922). • Adequately connected the three key variables of the PARIHS framework to the barriers and influences of getting evidence into practice. Issues: • Despite Strengths, 'the findings also suggest that further consideration is required to ensure that the PARIHS framework is appropriate, comprehensive, and accurate' (p. 921). • Criteria for inclusion and related meanings not always clear.	Strengths: • Presentation of findings was well organized and categorized by themes that emerged in the data. Issues: • Conclusions that findings confirm PARIHS did not seem adequately grounded. • No definitive a-priori measure of success and projects studied were complete yet. • Authors acknowledge study limitations as: 1) small sample sizes, 2) data credibility limited due to self-report, 3) potential bias as participants may have been 'evidence-based practice enthusiasts' (p. 920) and 4) successful implementation was 'defined largely by its absence than its presence' (p. 920) in the study.

Sharp	2004	Worldviews Evid Based Nurs	Strengths: • 'Desired outcomes can be achieved when the context is less than ideal but outcomes are generally poor when attention to both context and facilitation are lacking' (p. 137). • Authors learned the utility of PARIHS whereby new strategies can be developed. • Used as a diagnostic tool for retrospective study where interventions didn't work very well. Issues: • Variable definitions of elements; and variable placement of sub-elements.	Strengths: • Reinforced the importance of needs assessment of evidence, context and facilitation factors prior to the initiation of intervention implementation. • PARIHS model utilized to organize data and link empirical data to the model to demonstrate how it can inform real life situations. Issues: • Authors linked factors to outcomes globally, but not within sites, which would have helped understanding of the data, given the variable findings noted (there seemed to be an overlap of some barriers and facilitators).

Stetler	2006	Implement Sci	Strengths: • The study affirmed the importance of facilitation as a distinct role with a number of potentially crucial behaviors and activities. • Highlighted the importance of the task-oriented purpose. • Role of individual facilitator characteristics found to be important. Issues: • Categories under skills/attributes in PARIHS don't provide some of the details found in the study, nor does the framework adequately highlight the mixed facilitation approach found in primarily such task-oriented projects.	**Strengths**: • Use of a stimulated recall method gave interviewees several opportunities to continue recalling and adding to the richness of the qualitative data while further commenting, affirming or challenging the analysis **Issues**: • Authors noted the evaluation was 'both small scale and reliant on self-report data, thus potentially limiting its generalizability. Additionally, its purposively sampled participants represented a specific perspective and are likely EBP enthusiasts, particularly in terms of facilitation' (p. 12). • Only external facilitators were interviewed.

Wallin	2005	Int J Nurs Stud	Strengths: • Results support the role of the three main components (evidence, context, facilitation) in uptake of quality improvement initiatives. • Reasonable to use PARIHS to help frame discussion of findings. • Highlighted strong role of internal leadership. Issues: • Difficult to tease out sub/elements of PARIHS because of dynamic interrelationships between elements.	Strengths: • The only study that used PARIHS to frame results from a process evaluation within a randomized control trial. Issues: • PARIHS was used loosely as an organizing framework to present results and authors did not reflect back on utility of PARIHS.

Wallin	2006	Nurs Res	Strengths: • Results '...demonstrated empirical support for the validity of the context dimension of the PARIHS framework.' (p. 156) Showed a positive relationship between RU and context (culture, leadership, and evaluation) and further demonstrated a positive incremental relationship between RU and rank ordering of context from low to high. Issues: • Unclear implications for PARIHS definition of context, given how narrowly measured/defined out of unrelated dataset. • Unclear implications for definition of facilitation as it relates to inherent leader roles, such as a nurse manger.	Strengths: • Clear presentation of hypotheses and results. Issues: • RU was derived, in part, from contextual variables including autonomy and organizational slack, with rationale for doing so unclear. • Authors interpret results as validation for PARIHS but also recognize that 'only one of the PARIHS components - context - was used, and [they chose] only one variable to characterize each contextual dimension' (p. 158). • RU and context variables were selected, post hoc, from a dataset developed for another study.

### How and why PARIHS was used in studies

Empirical studies generally used PARIHS as an organizing framework for analyses, such as examining predictors of nurses' research utilization (RU) [9,10,34], or reporting findings, such as highlighting differences between a series of efficacy studies and a planned translational study [40] (Table 2 Overview of empirical articles included in the synthesis).

Stated reasons for using PARIHS included that it acknowledges the complexity of implementation (or knowledge translation) [39]; it includes contextual factors [38]; and that it explicitly includes and describes context and facilitation [30]. Generally, users referred to the intuitive appeal of the three main elements (evidence, context, and facilitation) and PARIHS's explicit acknowledgement of the complex interrelationships among elements and their effects on implementation. Five empirical articles provided no explicit rationale for selecting PARIHS.

### How PARIHS elements were operationalized

Three empirical papers described development of survey instruments based on PARIHS, two exclusively on the same survey assessing the element of context [35,36] and the other on evidence and context [11]. A series of three studies mapped survey items from secondary datasets to PARIHS elements, and tested their association with nurses' RU: one focused on context [34], and two on context and facilitation [9,10]. Except for a study by the PARIHS team [8], the empirical articles were not designed to validate or refine PARIHS.

Among non-quantitative empirical articles, two provided details of how PARIHS was operationalized: one specified questions used in a program evaluation [33], and another proposed a PARIHS-based framework to enhance reflective professional practice [41]. The nine remaining empirical articles did not specify how elements and sub-elements were measured or assessed, such as coding definitions or logic models for drawing conclusions about observed relationships.

### A critical appraisal of reviewed studies

A key strength of the existing PARIHS literature (Table 3 Core concept articles, and Table 4 Empirical articles) was that several studies used findings to comment on the framework in ways that could help refine or validate it. One example was a suggestion to address underlying motivation for change, such as relative advantage and tension for change [27,28]. Another was a qualitative exploration by the PARIHS team of how the framework fit with empirical findings [29]. A series of three articles attempted to quantify measures of context and facilitation and test quantitative multi-level models using facilitation and context as predictors of RU by nurses [9,10,34].

We identified two major issues with the PARIHS literature through our review. First, none of the studies used PARIHS prospectively to design implementation strategies. With the exception of articles reporting on survey development [11,35,36], all of the empirical studies were retrospective or cross-sectional. The six core concept papers described analyses that were conducted at a high level addressing broad concepts, and relied on non-systematic review of the literature.

Second, there was significant lack of detail about how variables were measured [39], mapped to PARIHS elements [38], or how results or conclusions were derived [33]. For example, Sharp and colleagues concluded that good implementation outcomes could be achieved in settings with poor context, but not both poor context and poor facilitation. However, the authors did not indicate which cases supported those conclusions and what characterized context and facilitation at those sites [30].

### A critical appraisal of the PARIHS framework

Several overarching strengths of PARIHS emerged (Table 3 Core concept articles, and Table 4 Empirical articles). First, though studies have not done so to date, the developers describe an explicit method for using PARIHS to guide diagnostic analysis of evidence and context [7], findings from which should be used to plan facilitation strategies to accomplish implementation.

Second are its flexibility and applicability to a range of settings, as well as perceptions by users that it captures key elements of the implementation experience. This includes PARIHS' expansive acknowledgement of what can and should constitute 'evidence,' and its recognition that implementation is a complex and multi-faceted process that is dynamic and often unpredictable. In additional, several articles reported findings that support specific PARIHS elements or sub-elements, such as Estabrooks and colleagues' finding that measures of facilitation and context are significantly associated with nurses' RU [10].

The primary issue related to the framework was a need for greater conceptual clarity about the definitions of sub-elements and the nature of dynamic relationships among elements and sub-elements. In many cases, sub-elements appear to have significant conceptual overlap. For example, criteria for evaluating receptive context include 'power and authority processes' and whether or not cultural boundaries are clearly defined and acknowledged. These two criteria appear to overlap with the culture and leadership sub-elements, which include being 'able to define culture(s) in terms of prevailing values/beliefs' and 'democratic inclusive decision making processes.' It is not clear what distinguishes receptive context, as a construct, from culture and leadership. Another example is that facilitation is defined solely as a role, and in terms of the individual who fills the role and the relationship they have with those implementing the change. As presently described, this element does not address implementation interventions such as reminders, web-based education, toolkits, social marketing, and audit and feedback that may be undertaken to facilitate implementation, and which could conceivably be untaken by a number of actors. Although PARIHS acknowledges the dynamic relationships among elements, the elements and sub-elements are described in linear terms, from 'low' to 'high,' with little explicit account of how or in what form dynamics among and across the sub-elements might emerge.

Both a strength and issue for PARIHS was the specification of the outcome 'successful implementation.' It was a strength in that the framework stipulates an outcome where many implementation models do not. However, there was little information in the six core articles about how to conceptualize or define successful implementation, and the empirical articles adopted a range of outcomes. Some articles used a broad outcome of RU [10,39], *i.e.*, the degree to which clinicians apply research knowledge in their practices generally. Others used the degree of implementation or uptake of specific practice changes [30,31].

## Discussion

Our objectives in the present synthesis were to understand how PARIHS has been used in implementation studies, how it has been operationalized, and the strengths and limitations of PARIHS and its supporting literature. We found a reasonably large published literature (33 published papers, 18 of which were empirical), but this is a body of findings that reflects many of the current limitations of the broader implementation science literature. These limitations provide great opportunities for improvement, notably three.

First, PARIHS was largely used and operationalized as an organizing device or heuristic, usually *post hoc*. However, PARIHS developers intended the framework to be used to assess evidence and context prior to implementation and then using these findings to guide facilitation of implementation. To move the framework forward, we need empirical studies that use PARIHS to prospectively design or comprehensively evaluate implementation activities. Researchers should explain the degree to which intervention design decisions and change strategies are based on PARIHS. The lack of prospective implementation studies is not unique to PARIHS; all but a fraction of published implementation studies fail to explicitly use any theory at all [43,44], so researchers do not appear to be conducting prospective implementation studies based on any conceptual frameworks; a similar lack of theoretical foundation is reported among studies of organizational factors linked to patient safety [45]. Our findings echo those of Kajermo and colleagues in a recent literature synthesis on use of the BARRIER scale, which is intended to prospectively identify barriers to research use by nurses [46]. Based on a paucity of prospective studies, they concluded that no further descriptive studies should be done, and that only prospective studies would move the science forward. We extend the same call for studies using PARIHS.

Second, though a strength of the empirical literature was that some studies showed empirical support for PARIHS, this finding needs to be interpreted in light of the overall study designs, which were retrospective case reports or cross-sectional analyses, and often lacked key methodological details. Furthermore, authors rarely contrasted findings to previous studies; the citation of prior work using PARIHS occurred almost exclusively in the introduction to set the stage for the study or conceptual rationale of the study. This too, may in part, be a function of the current development of the implementation science literature, and the natural evolution of standards and expectations about what details researchers most need to report. It may be time for something akin to CONSORT [47] or MOOSE [48] guidelines for reporting results of implementation intervention studies or implementation project evaluations. While implementation science may not be amenable to the same manner of checklists that have been applied to randomized trials and meta-analyses, there are key elements that could be described in sufficient specificity to provide guidance to both journal editors and researchers. These might include an explanation or rationale for mapping study findings to the constructs of the conceptual framework being used; a rationale for excluding certain elements; details about operationalization of constructs, including coding definitions for qualitative analyses; and discussion of the criteria authors use to draw conclusions about relationships between determinants and implementation outcomes. This might help address a key criticism of efforts to promote more theory-based implementation research, namely that translation of theory into intervention design is too subjective and opaque [49].

Finally, there are opportunities to improve the conceptual clarity of the framework itself, including refining conceptual definitions to more clearly draw distinctions among related sub-elements, such as receptive context, leadership, and culture. This will help provide for more rigorous studies by making it easier for users to map measures back to PARIHS consistently, derive testable hypotheses using the framework, and design more effective implementation strategies. We have drafted an implementation guide, being published separately, which discusses in more detail recommendations for those using PARIHS in task-oriented implementation projects and research, or seeking to refine the framework. Below, we briefly discuss three specific opportunities to refine the PARIHS framework.

First, PARIHS acknowledges the dynamic relationships among elements and sub-elements in the framework and the often unpredictable nature of implementation. However, dynamic implies that elements/sub-elements interact or act as modifiers or contingencies, such that the effects of one is dependent on others [50]. As a result, the same implementation intervention may have wildly different effects in different settings [51]. PARIHS would be strengthened even more by beginning to describe how those dynamics might emerge and provide examples that could eventually help identify more generalizable patterns. Identifying and describing all potential interactions is clearly impossible, but currently, PARIHS elements are described on a continuum, low to high, that strongly implies linear relationships, which are inconsistent both with the broader concept of PARIHS as a dynamic model and with available evidence. For example, we have prospective studies that find senior leadership support changes dramatically over time, with senior leaders shifting among roles ranging from institutional mentors for the change to critics of it [52]; and that senior leadership support is not always a strong driver and certainly not always a necessary condition for implementation [53,54]. It may be possible to identify generalizable contextual interactions, such as senior leadership support being necessary for EBPs that involve coordination across departments or services, require large capital investments or lack strong professional endorsement.

In part, the lack of specifics about interactions among elements may arise from PARIHS straddling the line between a higher order planned action (or prescriptive) theory (PAT) for use by change agents to guide their implementation strategy, and a classical (or descriptive/explanatory) model meant to describe or explain how change occurs. The core concept articles explicitly propose that PARIHS be used to guide implementation by assessing evidence and context in order to inform facilitation, strongly positioning PARIHS as a prescriptive model, albeit not with the detail of a PAT as described by Graham and Tetroe [53].

Second, we also noted that a more explicit definition for 'successful implementation' is needed. This again is both a key strength of the framework and an opportunity to strengthen it. A clear definition of successful implementation is critical for moving implementation science literature forward, and we may do well to draw on the literatures of other disciplines. For example, researchers in education [55] and health promotion [56] have written specifically about criteria for determining when new programs are fully implemented. Likewise, scholars in management have written about conceptual considerations for defining effective implementation of new practices such as IT systems [57] and banking practices [58], including distinguishing implementation from 'compliant' use that is either incomplete or likely to degrade.

Conceptually, successful implementation might comprise three distinct aspects, identified as part of our aforementioned implementation Guide. All represent seemingly necessary conditions for concluding that a project has achieved successful implementation: realization of the implementation plan or strategy; achievement and maintenance of the targeted EBP; and achievement and maintenance of end-point patient or organizational outcomes. These three components reflect a logic model linking an implementation strategy to ultimate outcomes. This definition of successful implementation affords an understanding of when and how an implementation program has delivered the benefits as hypothesized. To accomplish that, we need to assess whether the implementation strategy occurred as planned, whether the EBP was established as needed, and whether desired outcomes followed.

Third, other conceptual models should be drawn on and compared to better elaborate the core PARIHS elements or to better position work using PARIHS in the broader literature. The PARIHS core concept papers make it clear that the developers envision PARIHS being used in combination with other conceptual frameworks. Findings in some of the studies suggest the value of making additional attributes of the evidence-based change more explicit such as those identified in Rogers' Diffusion of Innovation framework [34]. For example, Rogers' innovation attribute of the observability of a new practice (*i.e.*, the extent to which its use by an individual is readily perceived by others in their social network) [2,59] does not appear to have an analogue in PARIHS. These types of comparisons and extensions would help build cumulative knowledge and inform refinements to the framework.

The PARIHS authors continue to revisit and refine the framework, recognize its limitations, and call for further research [7]. We consider a critical strength of any framework. Researchers [60] and practitioners [61] continue to use PARIHS and we expect more rigorous studies will be published. Already in the period since we completed our literature search, we are aware of at least five new publications citing PARIHS including two articles presenting results of validations of survey instruments based on the framework [62,63]. Also, several prospective research studies based on the framework are in progress by both the PARIHS team (http://www.parihs.org) and other research teams, including one conducting research in Vietnam and several conducting research in the Veterans Health Administration QUERI program within the US.

## Limitations

Our review had two limitations. First, we did not assess the 'gray' or unpublished literature or publications in languages other than English. In doing so, we may have missed important work relating to PARIHS.

Second, we focused exclusively on the PARIHS framework, and not on literature regarding other frameworks that may include similar or related constructs. Doing so was beyond the scope of our synthesis, though we do comment on the need for greater comparison and linkages between PARIHS and other frameworks.

Some may also view our methods as limited because we did not conduct a quantitative meta-analysis. However, we used methods appropriate to our research questions and to the literature being reviewed, which included few quantitative studies. We also took several steps to increase the transparency and reliability of our results.

## Summary

The single greatest need for researchers using PARIHS, and other implementation models, is to use the framework prospectively and comprehensively, and evaluate that use relative to its perceived strengths and issues for enhancing successful implementation. Ultimately, the proof of any implementation framework is its demonstrated usefulness in practical terms to design implementation interventions and make implementation more effective under various conditions. Studies using the framework in this way will move the whole field forward.

Researchers using PARIHS in studies or to guide action research should clearly explain how PARIHS is used and how interventions or measures map to specific PARIHS elements. For example, studies of facilitation activities should explain how facilitation purpose, role and skills and attributes were defined or taken into account. Other reviews have similarly called for more explicit and detailed explanation of how theory is used in implementation studies [43,44]. It may be time for the implementation science community to develop consensus guidelines for what should be reported.

## Competing interests

The authors declare that they have no competing interests.

## Authors' contributions

CBS conceived the study. All authors abstracted, reviewed data and provided critical input on findings. CDH wrote first draft of paper and CBS, LJD and HH provided major input and revisions. All authors read, critiqued and approved the final manuscript.

## Supplementary Material

Additional file 1 **Synopsis template**. The synopsis template is a semi-structured form for initial narrative abstraction and critique of the included articles. It included the article abstract and six sections to be filled out by the reviewer, such as aspects of the PARIHS framework said to influence the study. Click here for file

Additional file 2 **Summary table template for empirical articles**. The summary table template is a semi-structured tool for article abstraction and critique that was in tabular format and included more discrete data elements than the synopsis template, *e.g.*, broken down by PARIHS element and sub-element. The summary table differed between the core-concept and empirical articles because of the types of publication (*e.g.*, differences in the purposes and methods of the papers). This is the summary table for the empirical articles. Click here for file

Additional file 3 **Summary table template for core concept articles**. The summary table template is a semi-structured tool for article abstraction and critique that was in tabular format and included more discrete data elements than the synopsis template, *e.g.*, broken down by PARIHS element and sub-element synthesis. The summary table differed between the core-concept and empirical articles because of the types of publication and related content (*e.g.*, differences in the purposes and methods of the papers). This is the summary table for the core concept articles. Click here for file

Additional file 4 **Commentaries excluded from the synthesis**. This is a table of eight papers that were reviewed as part of our literature review and ultimately excluded because we defined them as commentaries that neither presented empirical research related to PARIHS nor conceptual critique or elaboration of the framework. The table includes abstracted data on the purpose of paper; the rationale for using PARIHS; and how PARIHS was to be used. Click here for file

## References

[B1] McGlynn EA Asch SM Adams J Keesey J Hicks J DeCristofaro A Kerr EA The quality of health care delivered to adults in the United States N Engl J Med 2003 348 26 2635 2645 10.1056/NEJMsa022615 12826639

[B2] Damschroder LJ Aron DC Keith RE Kirsh SR Alexander JA Lowery JC Fostering implementation of health services research findings into practice: a consolidated framework for advancing implementation science Implement Sci 2009 4 50 10.1186/1748-5908-4-50 19664226 PMC2736161

[B3] Kitson A Harvey G McCormack B Enabling the implementation of evidence based practice: a conceptual framework Quality in Health Care 1998 7 3 149 158 10.1136/qshc.7.3.149 10185141 PMC2483604

[B4] Harvey G Loftus-Hills A Rycroft-Malone J Titchen A Kitson A McCormack B Seers K Getting evidence into practice: the role and function of facilitation Journal of Advanced Nursing 2002 37 6 577 588 10.1046/j.1365-2648.2002.02126.x 11879422

[B5] McCormack B Kitson A Harvey G Rycroft-Malone J Titchen A Seers K Getting evidence into practice: the meaning of 'context' J Adv Nurs 2002 38 1 94 104 10.1046/j.1365-2648.2002.02150.x 11895535

[B6] Rycroft-Malone J Seers K Titchen A Harvey G Kitson A McCormack B What counts as evidence in evidence-based practice? J Adv Nurs 2004 47 1 81 90 10.1111/j.1365-2648.2004.03068.x 15186471

[B7] Kitson A Rycroft-Malone J Harvey G McCormack B Seers K Titchen A Evaluating the successful implementation of evidence into practice using the PARiHS framework: theoretical and practical challenges Implementation Science 2008 3 1 1 10.1186/1748-5908-3-1 18179688 PMC2235887

[B8] Stetler CB Legro MW Rycroft-Malone J Bowman C Curran G Guihan M Hagedorn H Pineros S Wallace CM Role of "external facilitation" in implementation of research findings: a qualitative evaluation of facilitation experiences in the Veterans Health Administration Implement Sci 2006 1 23 10.1186/1748-5908-1-23 17049080 PMC1635058

[B9] Cummings GG Estabrooks CA Midodzi WK Wallin L Hayduk L Influence of organizational characteristics and context on research utilization Nursing research 2007 56 4 Suppl S24 39 10.1097/01.NNR.0000280629.63654.95 17625471

[B10] Estabrooks CA Midodzi WK Cummings GG Wallin L Predicting research use in nursing organizations: a multilevel analysis Nursing research 2007 56 4 Suppl S7 23 10.1097/01.NNR.0000280647.18806.98 17625477

[B11] Bahtsevani C Willman A Khalaf A Östman M Developing an instrument for evaluating implementation of clinical practice guidelines: a test-retest study Journal of Evaluation in Clinical Practice 2008 14 5 839 846 18331325 10.1111/j.1365-2753.2007.00916.x

[B12] Eccles M Grimshaw J Walker A Johnston M Pitts N Changing the behavior of healthcare professionals: the use of theory in promoting the uptake of research findings Journal of Clinical Epidemiology 2005 58 107 112 10.1016/j.jclinepi.2004.09.002 15680740

[B13] Grol RPTM Bosch MC Hulscher MEJL Eccles MP Wensing M Planning and Studying Improvement in Patient Care: The Use of Theoretical Perspectives The Milbank Quarterly 2007 85 1 93 138 10.1111/j.1468-0009.2007.00478.x 17319808 PMC2690312

[B14] ICEBeRG TICEtBR Group Designing theoretically-informed implementation interventions Implementation Science 2006 1 1 4 10.1186/1748-5908-1-4 16722571 PMC1436012

[B15] Rycroft-Malone J Kitson A Harvey G McCormack B Seers K Titchen A Estabrooks C Ingredients for change: revisiting a conceptual framework Qual Saf Health Care 2002 11 2 174 180 10.1136/qhc.11.2.174 12448812 PMC1743587

[B16] Kitson AL Rycroft-Malone J Harvey G McCormack B Seers K Titchen A Evaluating the successful implementation of evidence into practice using the PARiHS framework: theoretical and practical challenges Implementation Science 2008 3 1 1 10.1186/1748-5908-3-1 18179688 PMC2235887

[B17] Sandelowski M Barroso J Handbook for synthesizing qualitative research 2007 New York: Springer Pub. Co

[B18] Larkin RM Challenges to prison-based mental health research: a case study D.N.Sc 2008 Columbia University

[B19] Donaldson NE Rutledge DN Ashley J Outcomes of adoption: measuring evidence uptake by individuals and organizations Worldviews on evidence-based nursing/Sigma Theta Tau International, Honor Society of Nursing 2004 1 Suppl 1 S41 51 10.1111/j.1524-475X.2004.04048.x 17129334

[B20] Kavanagh T Stevens B Seers K Sidani S Watt-Watson J Examining Appreciative Inquiry as a knowledge translation intervention in pain management Canadian Journal of Nursing Research 2008 40 2 40 56 18714897

[B21] Kavanagh T Watt-Watson J Stevens B An examination of the factors enabling the successful implementation of evidence-based acute pain practices into pediatric nursing Children's Health Care 2007 36 3 303 321

[B22] Larkin ME Griffith CA Capasso VA Cierpial C Gettings E Walsh K O'Malley C Promoting research utilization using a conceptual framework J Nurs Adm 2007 37 11 510 516 10.1097/01.NNA.0000295617.26980.d1 17975468

[B23] O'Halloran P Martin G Connolly D A model for developing, implementing, and evaluating a strategy to improve nursing and midwifery care Practice Development in Health Care 2005 4 4 180 191 10.1002/pdh.20

[B24] Rycroft-Malone J The PARIHS framework--a framework for guiding the implementation of evidence-based practice Journal of nursing care quality 2004 19 4 297 304 15535533 10.1097/00001786-200410000-00002

[B25] Wallin L Profetto-McGrath J Levers MJ Implementing nursing practice guidelines: a complex undertaking J Wound Ostomy Continence Nurs 2005 32 5 294 300 discussion 300-291 16234720 10.1097/00152192-200509000-00004

[B26] Walsh K Lawless J Moss C Allbon C The development of an engagement tool for practice development Practice Development in Health Care 2005 4 124 130 10.1002/pdh.7

[B27] Ellis I Howard P Larson A Robertson J From workshop to work practice: An exploration of context and facilitation in the development of evidence-based practice Worldviews on evidence-based nursing/Sigma Theta Tau International, Honor Society of Nursing 2005 2 2 84 93 10.1111/j.1741-6787.2005.04088.x 17040545

[B28] Owen S Milburn C Implementing research findings into practice: improving and developing services for women with serious and enduring mental health problems J Psychiatr Ment Health Nurs 2001 8 3 221 231 10.1046/j.1365-2850.2001.00390.x 11882131

[B29] Rycroft-Malone J Harvey G Seers K Kitson A McCormack B Titchen A An exploration of the factors that influence the implementation of evidence into practice J Clin Nurs 2004 13 8 913 924 10.1111/j.1365-2702.2004.01007.x 15533097

[B30] Sharp ND Pineros SL Hsu C Starks H Sales AE A Qualitative Study to Identify Barriers and Facilitators to Implementation of Pilot Interventions in the Veterans Health Administration (VHA) Northwest Network Worldviews on evidence-based nursing/Sigma Theta Tau International, Honor Society of Nursing 2004 1 2 129 139 10.1111/j.1741-6787.2004.04023.x 17129326

[B31] Stetler C Legro M Rycroft-Malone J Bowman C Curran G Guihan M Hagedorn H Pineros S Wallace C Role of "external facilitation" in implementation of research findings: a qualitative evaluation of facilitation experiences in the Veterans Health Administration Implementation Science 2006 1 1 23 10.1186/1748-5908-1-23 17049080 PMC1635058

[B32] Wallin L Rudberg A Gunningberg L Staff experiences in implementing guidelines for Kangaroo Mother Care--a qualitative study Int J Nurs Stud 2005 42 1 61 73 10.1016/j.ijnurstu.2004.05.016 15582640

[B33] Conklin J Stolee P A model for evaluating knowledge exchange in a network context The Canadian journal of nursing research = Revue canadienne de recherche en sciences infirmieres 2008 40 2 116 124 18714901

[B34] Wallin L Estabrooks CA Midodzi WK Cummings GG Development and validation of a derived measure of research utilization by nurses Nursing research 2006 55 3 149 160 10.1097/00006199-200605000-00001 16708039

[B35] Wright J Developing a tool to assess person-centred continence care Nurs Older People 2006 18 6 23 28 16878809

[B36] Wright J McCormack B Coffey A McCarthy G Evaluating the context within which continence care is provided in rehabilitation units for older people INTERNATIONAL JOURNAL OF OLDER PEOPLE NURSING 2007 2 1 9 19 10.1111/j.1748-3743.2007.00046.x 20925827

[B37] Brown D McCormack B Developing Postoperative Pain Management: Utilising the Promoting Action on Research Implementation in Health Services (PARIHS) Framework Worldviews on Evidence-Based Nursing 2005 2 3 131 141 10.1111/j.1741-6787.2005.00024.x 17040534

[B38] Meijers JM Janssen MA Cummings GG Wallin L Estabrooks CA R YGH Assessing the relationships between contextual factors and research utilization in nursing: systematic literature review Journal of advanced nursing 2006 55 5 622 635 10.1111/j.1365-2648.2006.03954.x 16907795

[B39] Milner M Estabrooks CA Myrick F Research utilization and clinical nurse educators: A systematic review Journal of evaluation in clinical practice 2006 12 6 639 655 10.1111/j.1365-2753.2006.00632.x 17100863

[B40] Alkema GE Frey D Implications of translating research into practice: a medication management intervention Home health care services quarterly 2006 25 1-2 33 54 10.1300/J027v25n01_03 16803737

[B41] Doran DM Sidani S Outcomes-focused knowledge translation: a framework for knowledge translation and patient outcomes improvement Worldviews on evidence-based nursing/Sigma Theta Tau International, Honor Society of Nursing 2007 4 1 3 13 10.1111/j.1741-6787.2007.00073.x 17355405

[B42] McCormack B McCarthy G Development of the Context Assessment Index (CAI) 2008 Republic of Ireland Health Research Board and the Northern Ireland Department of Health, Social Services and Public Safety

[B43] Davies P Walker A Grimshaw J A systematic review of the use of theory in the design of guideline dissemination and implementation strategies and interpretation of the results of rigorous evaluations Implementation Science 2010 5 1 14 10.1186/1748-5908-5-14 20181130 PMC2832624

[B44] Davies P Walker A Grimshaw J Theories of behavior change in studies of guideline implementation Proc Br Psychol Soc 2003 11

[B45] Hoff T Jameson L Hannan E Flink E A review of the literature examining linkages between organizational factors, medical errors, and patient safety Med Care Res Rev 2004 61 1 3 37 10.1177/1077558703257171 15035855

[B46] Nilsson Kajermo K Bostrom AM Thompson D Hutchinson A Estabrooks C Wallin L The BARRIERS scale -- the barriers to research utilization scale: A systematic review Implementation Science 2010 5 1 32 10.1186/1748-5908-5-32 20420696 PMC2883534

[B47] Schulz KF Altman DG Moher D CONSORT 2010 Statement: updated guidelines for reporting parallel group randomised trials BMC Med 2010 8 18 10.1186/1741-7015-8-18 20334633 PMC2860339

[B48] Stroup DF Berlin JA Morton SC Olkin I Williamson GD Rennie D Moher D Becker BJ Sipe TA Thacker SB Meta-analysis of observational studies in epidemiology: a proposal for reporting. Meta-analysis Of Observational Studies in Epidemiology (MOOSE) group Jama 2000 283 15 2008 2012 10.1001/jama.283.15.2008 10789670

[B49] Bhattacharyya O Reeves S Garfinkel S Zwarenstein M Designing theoretically-informed implementation interventions: Fine in theory, but evidence of effectiveness in practice is needed Implementation Science 2006 1 1 5 10.1186/1748-5908-1-5 16722583 PMC1436014

[B50] Johns G The essential impact of context on organizational behavior Academy of Management Review 2006 31 2 386 408

[B51] Helfrich CD Weiner BJ McKinney MM Minasian L Determinants of Implementation Effectiveness: Adapting a Framework for Complex Innovations Med Care Res Rev 2007 64 3 279 303 10.1177/1077558707299887 17507459

[B52] Van De Ven AH Polley DE The Innovation Journey 1999 New York: Oxford University Press

[B53] Edmondson A Psychological Safety and Learning Behavior in Work Teams Administrative Science Quarterly 1999 44 2 350 383 10.2307/2666999

[B54] Edmondson A Speaking up in the operating room: How team leaders promote learning in interdisciplinary action teams Journal of Management Studies 2003 40 1419 1452 10.1111/1467-6486.00386

[B55] Yin RK Changing urban bureaucracies: How new practices become routinized 1979 Lexington, MA: Lexington Books

[B56] Goodman RM Steckler A A model for the institutionalization of health promotion programs Family & Community Health 1989 11 4 63 78

[B57] Klein KJ Sorra JS The challenge of innovation implementation Academy of Management Review 1996 21 4 1055 1080 10.2307/259164

[B58] Nord WR Tucker S Implementing Routine and Radical Innovations 1987 Lexington, Massachusetts: Lexington Books

[B59] Rogers EM Diffusion of Innovations 2003 Fifth New York, NY: The Free Press

[B60] Rycroft-Malone J Fontenla M Seers K Bick D Protocol-based care: the standardisation of decision-making? Journal of Clinical Nursing 2009 18 10 1490 1500 10.1111/j.1365-2702.2008.02605.x 19413539

[B61] Capasso V Collins J Griffith C Lasala CA Kilroy S Martin AT Pedro J Wood SL Outcomes of a clinical nurse specialist-initiated wound care education program: using the promoting action on research implementation in health services framework Clinical Nurse Specialist: The Journal for Advanced Nursing Practice 2009 23 5 252 257 10.1097/NUR.0b013e3181b207f5 19710571

[B62] McCormack B McCarthy G Wright J Coffey A Development and Testing of the Context Assessment Index (CAI) Worldviews on Evidence-Based Nursing 2009 6 1 27 35 10.1111/j.1741-6787.2008.00130.x 19207560

[B63] Helfrich CD Li YF Sharp ND Sales AE Organizational readiness to change assessment (ORCA): Development of an instrument based on the Promoting Action on Research in Health Services (PARiHS) framework Implementation Science 2009 4 1 38 10.1186/1748-5908-4-38 19594942 PMC2716295

