# Supplementary material for: A critical synthesis of literature on the promoting action on research implementation in health services (PARIHS) framework
Source: Implement Sci. 2010 Oct 25;5:82. doi: 10.1186/1748-5908-5-82 (PMC2988065; doi:10.1186/1748-5908-5-82)
Supplement: Additional file 1 — Synopsis template. The synopsis template is a semi-structured form for initial narrative abstraction and critique of the included articles. It included the article abstract and six sections to be filled out by the reviewer, such as aspects of the PARIHS framework said to influence the study. [file 1748-5908-5-82-S1.DOC]

METHODS FOR LITERATURE SYNOPSIS/CRITIQUES FOR USER FRIENDLY GUIDE TO THE PARIHS FRAMEWORK

**Review by:__________________**

**Citation**

1. **Written Product to be Submitted after Presentation/Discussion of Review at *PDI* meeting: Format as follows:**
   1. ***PubMed Abstract***
   2. ***Key Points* (see below)**
2. **Key Points:**
   1. **Conceptual articles:**
      1. **If any, major concerns about the quality of the conceptual work.**
      2. **A summary of how the authors defined the framework:**
         - **The meaning of major aspects and elements of the framework, and the boundaries, if any, they placed around each to help operationalization.**
         - **The expected or substantiated nature of the relationship among the core elements, in variable circumstances, and the related degree of success.**
         - **Substantiating evidence re: the framework.**
      3. **Critique of the framework by the authors**
      4. **A critique of the framework by the reviewer, e.g.:**
         - **What does the framework or individual elements add to our understanding of “successful implementation”**
         - **What components of elements/sub-elements appear to have broad applicability?**
         - **How might the frameworks be used within various studies?**
         - **How might the framework be strengthened?**

- - - - **Draft hypotheses, propositions, and/or questions for discussion**
  1. ***Recommendations from PDI discussion*.**
