# Supplementary material for: A critical synthesis of literature on the promoting action on research implementation in health services (PARIHS) framework
Source: Implement Sci. 2010 Oct 25;5:82. doi: 10.1186/1748-5908-5-82 (PMC2988065; doi:10.1186/1748-5908-5-82)
Supplement: Additional file 2 — Summary table template for empirical articles. The summary table template is a semi-structured tool for article abstraction and critique that was in tabular format and included more discrete data elements than the synopsis template, e.g., broken down by PARIHS element and sub-element. The summary table differed between the core-concept and empirical articles because of the types of publication (e.g., differences in the purposes and methods of the papers). This is the summary table for the empirical articles. [file 1748-5908-5-82-S2.DOC]

| **CRITIQUE by [reviewer name]: [full article citation]**  ABSTRACT: [Article abstract] | | | | | |
| --- | --- | --- | --- | --- | --- |
| **OVERALL METHODS** | **Major concerns RE: quality of study** | **Outcomes of the study [H’d & achieved]** | **How PARIHS was “used”** | | **Congruency between model & design** |
|  |  |  | **PROPOSED:** | **ACTUAL:** |  |
| **EVIDENCE** | **How defined/inclusion** | **How defined/exclusion** | **How measured** | **What was learned?[[1]](#footnote-2)** | **Barriers/enablers for success including related circumstances[[2]](#footnote-3)** |
| **ASTERISK all sub-elements “USED”; again use quotes, prn** |  |  |  |  |  |
| ***Research*** |  |  |  |  |
|  |  |  |  |  |
| ***Clinical experience*** |  |  |  |  |
|  |  |  |  |  |
| ***Patient experience*** |  |  |  |  |
|  |  |  |  |  |
| ***Information/data from local context*** |  |  |  |  |
|  |  |  |  |  |
| **CONTEXT** | **How defined/inclusion** | **How defined/exclusion** | **How measured** | **What was learned?** | **Barriers/enablers for success including related circumstances** |
|  |  |  |  |  |  |
| ***Receptive context*** |  |  |  |  |  |
|  |  |  |  |  |  |
| ***Culture*** |  |  |  |  |  |
|  |  |  |  |  |  |
| ***Leadership*** |  |  |  |  |  |
|  |  |  |  |  |  |
| ***Evaluation*** |  |  |  |  |  |
|  |  |  |  |  |  |
| **FACILITATION[[3]](#footnote-4)** | **How defined/inclusion** | **How defined/exclusion** | **How measured** | **What was learned?** | **Barriers/enablers for success including related circumstances** |
|  |  |  |  |  |  |
| ***Purpose*** |  |  |  |  |  |
|  |  |  |  |  |  |
| ***Role*** |  |  |  |  |  |
|  |  |  |  |  |  |
| ***Skill & attributes*** |  |  |  |  |  |
|  |  |  |  |  |  |
| **RELATIONSHIPS** | **How defined/inclusion** | **How defined/exclusion** | **How measured** | **What was learned?** | **Barriers/enablers for success including related circumstances** |
|  |  |  |  |  |  |
|  |  |  |  |  |  |
|  |  |  |  |  |  |
|  |  |  |  |  |  |
|  |  |  |  |  |  |
|  |  |  |  |  |  |
|  |  |  |  |  |  |
| **OTHER** | **What was learned?** | **Questions re: framework** | **Posed Hs** | **Suggested methods** | **Other observations/recommendations of reviewer or authors** |
|  |  |  |  |  |  |
|  |  |  |  |  |  |
|  |  |  |  |  |  |

1. Including about the value of the “use” of the framework [↑](#footnote-ref-2)
2. E.g., type of setting and innovation [↑](#footnote-ref-3)
3. Relative to this element, sub elements not clear in the Appendix in 2008; thus used 2004 document [↑](#footnote-ref-4)
