# Supplementary material for: A critical synthesis of literature on the promoting action on research implementation in health services (PARIHS) framework
Source: Implement Sci. 2010 Oct 25;5:82. doi: 10.1186/1748-5908-5-82 (PMC2988065; doi:10.1186/1748-5908-5-82)
Supplement: Additional file 3 — Summary table template for core concept articles. The summary table template is a semi-structured tool for article abstraction and critique that was in tabular format and included more discrete data elements than the synopsis template, e.g., broken down by PARIHS element and sub-element synthesis. The summary table differed between the core-concept and empirical articles because of the types of publication and related content (e.g., differences in the purposes and methods of the papers). This is the summary table for the core concept articles. [file 1748-5908-5-82-S3.DOC]

| **CRITIQUE by [reviewer name]: [full article citation]**  ABSTRACT: [Article abstract] | | | | | | |
| --- | --- | --- | --- | --- | --- | --- |
| **Evidence** | **Definition:**  **Inclusion** | **Definition:**  **Exclusion** | **Strengths** | **Limitations** | **Other observations** | **Recommendations** |
|  |  |  |  |  |  |  |
| ***Research*** |  |  |  |  |  |  |
| ***Practice / Clinical Experience*** |  |  |  |  |  |  |
| ***Patient Experience*** |  |  |  |  |  |  |
| ***Routine Data*** |  |  |  |  |  |  |
|  |  |  |  |  |  |  |
| **CONTEXT** | **Definition:**  **Inclusion** | **Definition:**  **Exclusion** | **Strengths** | **Limitations** | **Other observations** | **Recommendations** |
| ***Leadership*** |  |  |  |  |  |  |
| ***Receptive context*** |  |  |  |  |  |  |
| ***Culture*** |  |  |  |  |  |  |
| ***Measurement/ Evaluation*** |  |  |  |  |  |  |
| **FACILITATION** | **Definition:**  **Inclusion** | **DEFINITION**  **EXCLUSION** | **Strengths** | **Limitations** | **Other observations** | **Recommendations** |
|  |  |  |  |  |  |  |
| **OTHER** | **Definition:**  **Inclusion** | **Definition:**  **Exclusion** | **Strengths** | **Limitations** | **Other observations** | **Recommendations** |
|  |  |  |  |  |  |  |
| **OVERALL** |  |  |  |  |  |  |
