# Supplementary material for: A critical synthesis of literature on the promoting action on research implementation in health services (PARIHS) framework
Source: Implement Sci. 2010 Oct 25;5:82. doi: 10.1186/1748-5908-5-82 (PMC2988065; doi:10.1186/1748-5908-5-82)
Supplement: Additional file 4 — Commentaries excluded from the synthesis. This is a table of eight papers that were reviewed as part of our literature review and ultimately excluded because we defined them as commentaries that neither presented empirical research related to PARIHS nor conceptual critique or elaboration of the framework. The table includes abstracted data on the purpose of paper; the rationale for using PARIHS; and how PARIHS was to be used. [file 1748-5908-5-82-S4.DOC]

Additional File 4: Commentaries excluded from the synthesis

| **Author** | **Year** | **Journal** | **Method** | **Sample** | **Purpose of study / paper** | **Rationale for using PARIHS** | **How PARIHS used** |
| --- | --- | --- | --- | --- | --- | --- | --- |
| Donaldson | 2004 | Worldviews Evid Based Nurs | Commentary | Not applicable | Explore the issue of measuring outcomes regarding KU. | One of 4 theories cited as having implications for measuring outcomes and uptake of innovation. | Didn’t “use” PARIHS per se; rather commented on it in referencing frameworks that have at least a suggestion about importance of outcomes. |
| Kavanaugh | 2007 | Child Health Care | Commentary | Not applicable | Examine factors contributing to effective implementation of evidence-based acute pain practices in pediatric nursing. | PARIHS approaches implementation with "greater depth and more comprehensively relative to other [models]."  Schein's framework on organizational culture also used, and Rogers' DOI cited. | Appeared to be an organizing tool for the paper, per use of PARIHS elements/sub-elements for section headings/sub- headings; but without other, integrated, substantive use. |
| Kavanaugh | 2008 | Can J Nurs Res | Commentary | Not applicable | Establish Appreciative Inquiry (AI) as a knowledge translation intervention for nursing pain management. | No explicit rationale provided. | To validate *Appreciative Inquiry* as a potential theory-based knowledge translation intervention. |
| Larkin | 2007 | J Nurs Adm | Commentary | Not applicable | Describe activities of a Nursing Research Committee to promote research and evidence-based practice in nursing. | No explicit rationale, though PARIHS is presented as having been tested, with the implication that it provides a validated framework. | PARIHS elements described in the background, particularly context, but are not linked to the nursing research committee activities. |
| O'Halloran | 2005 | Practice Development in Health Care | Commentary | Acute hospital trust in Northern Ireland | Describe a structured process for developing and implementing a *strategic plan* [rather than EBP project] for nursing and midwifery (including values clarification, critical companionship and focus groups). | Implicitly because PARIHS identifies facilitation and “emancipatory” leadership styles as key factors in successful implementation. | Used a modification of PARIHS, but it was not clear how the model was modified; sub-elements not referenced at all. |
| Rycroft-Malone | 2004 | J Nurse Care Q | Commentary | Not applicable | “Present practical information for direct-care nurses and quality improvement leaders about using the best available evidence to change practice.” | Not applicable. | Focus of paper. |
| Wallin | 2005 | J Wound Ostomy Continence Nurs | Commentary | Not applicable | Discuss implementation of clinical practice. | Not applicable. | As a framework for a discussion of implementation of evidence based practices. |
| Walsh | 2005 | Practice Development in Health Care | Commentary | Not applicable | Develop a guiding tool to help clinicians “engage” in practice change. | PARIHS “outlined collaborative ways of working, principles of active facilitation and the incorporation of evidence in bringing about evidence-based practice change through the implementation of research evidence into practice." | Their engagement tool was organized relative to the 3 core elements (E,C,F); included only limited discussion of sub-elements). |
